# Supplementary material for: Maternal and Dietary Factors Are Associated with Metabolic Syndrome in Women with a Previous History of Gestational Diabetes Mellitus
Source: Int J Environ Res Public Health. 2022 Dec 14;19(24):16797. doi: 10.3390/ijerph192416797 (PMC9779785; doi:10.3390/ijerph192416797)
Supplement: Supplementary file 1 [file ijerph-19-16797-s001.zip › ijerph-2040148-supplementary.pdf]

Table S1. Factor loading scores of dietary patterns identified by principal component analysis

Food group	Factor loading score of dietary patterns	
	**Western Dietary Pattern**	**Prudent Dietary Pattern**
Cereals and grains	0.49	0.236
Fast food	0.692	0.091
Meat and eggs	-0.047	0.462
Fish and seafood	-0.069	0.611
Legumes	0.086	0.298
Milk and dairy products	0.162	0.415
Fruits and vegetables	0.152	0.686
Coffee, tea and sugar-sweetened beverages	0.471	0.041
Confectionaries	0.449	0.182
Sugar, spreads and creamer	0.708	-0.263
Salty food and condiments	0.735	0.055
